# Effect of different interventions for latent tuberculosis infections in China: a model-based study

**DOI:** 10.1186/s12879-022-07465-5

**Published:** 2022-05-23

**Authors:** Zexuan Wen, Tao Li, Wenlong Zhu, Wei Chen, Hui Zhang, Weibing Wang

**Affiliations:** 1 Department of Epidemiology, School of Public Health, Fudan University, Shanghai, 200032 China; 2 Key Laboratory of Public Health Safety of Ministry of Education, Fudan University, Shanghai, 200032 China; 3 National Center for Tuberculosis Control and Prevention, Chinese Center for Disease Control and Prevention, Beijing, 100050 China; 4 Shanghai Institute of Infectious Disease and Biosecurity, Fudan University, Shanghai, 200032 China

**Keywords:** Tuberculosis, SEIR model, Preventive treatment, Screen, Intervention strategy, Active case finding

## Abstract

**Background:**

Tuberculosis (TB) has a serious impact on people’s health. China is one of 30 countries that has a high TB burden. As the currently decreasing speed of the incidence of TB, the WHO’s goal of “End TB Strategy” is hard to achieve by 2035. As a result, a SEIR model that determines the impact of different tuberculosis preventive treatments (TPTs) in different age groups, and the effect of different interventions on latent TB infections (LTBIs) in China is developed.

**Methods:**

A Susceptible-Exposed-Infectious-Recovered (SEIR) model was established. Goodness-of-fit tests were used to assess model performance. Predictive analysis was used to assess the effect of different interventions on LTBIs and achieving the goals of the “End TB Strategy”.

**Results:**

The Chi-square test indicated the model provided a good statistical fit to previous data on the incidence of TB (χ^2^ = 0.3085, p > 0.999). The 1HP treatment regimen (daily rifapentine + isoniazid for 4 weeks) was most effective in reducing the number of TB cases by 2035. The model indicated that several strategies could achieve the 2035 target of the “End TB Strategy”: completion of active case finding (ACF) for LTBI and TPT nation-wide within 5 years; completion of ACF for LTBIs and TPT within 2 years in high-incidence areas; completion of TPT in the elderly within 2 years; or introduction of a new vaccine in which the product of annual doses and vaccine efficiency in the three age groups above 14 years old reached 10.5 million.

**Conclusion:**

The incidence of TB in China declined gradually from 2005 to 2019. Implementation of ACF for LTBIs and TPT nation-wide or in areas with high incidence, in the elderly, or administration of a new and effective vaccine could greatly reduce the number of TB cases and achieve the 2035 target of the “End TB Strategy” in China.

**Supplementary Information:**

The online version contains supplementary material available at 10.1186/s12879-022-07465-5.

## Background

Tuberculosis (TB), an infectious disease caused by *Mycobacterium tuberculosis* (Mtb), is a significant public health problem worldwide. China is one of 30 countries that has a high TB burden. During 2020, China had 842,000 new cases of TB, and was only surpassed by India [1]. The 2020 Health Statistics Yearbook reported the incidence of pulmonary TB in China was 55.55 per 100,000 people [2], second in the ranking of class A and B notifiable infectious diseases. The World Health Organization (WHO) proposed the “End TB Strategy” in 2014, and this included a series of targets [3]. However, based on the current annual rate of decline in the incidence of TB [4] (1–2%), these WHO targets may not be achieved. At the same time, the COVID-19 pandemic disrupted the worldwide tuberculosis response, and the progress in reducing TB disease burden tended to be reversed [1]. It is estimated that the number of TB cases in China will be even higher in 2020 than in 2019 [1, 4], and the impact of COVID-19 on incidence will be biggest in 2022 [1]. To mitigate and reverse the impacts, restoring access to and provision of essential TB services is the immediate priority.

In addition to active TB, many individuals have latent TB infections (LTBIs), in which Mtb is undetectable. In these individuals, the body’s immune system controls the infection and prevents the development of active TB. Individuals with LTBIs have no clinical symptoms and cannot transmit the bacteria to others [5]. Nevertheless, 5 to 10% of people infected with Mtb have a risk of developing active TB, especially within 5 years of the initial infection [6], and this percentage is even higher among those with immunosuppressive diseases [7].

There are now about 360-million individuals with Mtb infections in China [8], most of which are LTBIs [6]. Epidemiological surveys have shown that 85% to 90% of newly diagnosed active TB evolves from LTBIs with a positive tuberculin test [9], making prevention and treatment more difficult. Screening and preventive treatment of high-risk groups in China may help reduce the incidence of TB. In fact, recent screening and preventive interventions that targeted people with LTBIs in China have achieved some success [4, 10, 11]. However, due to the unique characteristics of the TB epidemic and the lack of evidence-based results regarding the effect of preventive measures, the effects of different interventions are uncertain. Based on previous studies that developed dynamic models of TB [10–14], we modeled the combined effects of a large-scale TB preventive treatment (TPT) with traditional interventions, and examined the effects of vaccination and active case-finding (ACF) for LTBIs on the incidence of TB in China (ACF in this study denotes active screening and finding LTBIs in the entire population).

## Methods

### Data collection

Demographic data from the National Bureau of Statistics, including the number of individuals in different age groups at different times [2], were converted into parameters of corresponding time units for construction of the model.

All data on TB incidence were from the Infectious Diseases Reporting System (IDRS) of the Chinese Bureau of Disease Control. Complete monthly and annual data were available from 2005 to 2020. The TB Information Management System (TBIMS) was examined to obtain data on patients diagnosed with TB from 2008 to 2018 for parameter acquisition and model construction. These data included statistical reports, with classification by gender, age, occupation, key event time (including the time of diagnosis, start of treatment, registration time, etc.), region of residence, treatment outcome, and drug resistance.

### Model formulation

**Fig. 1:**
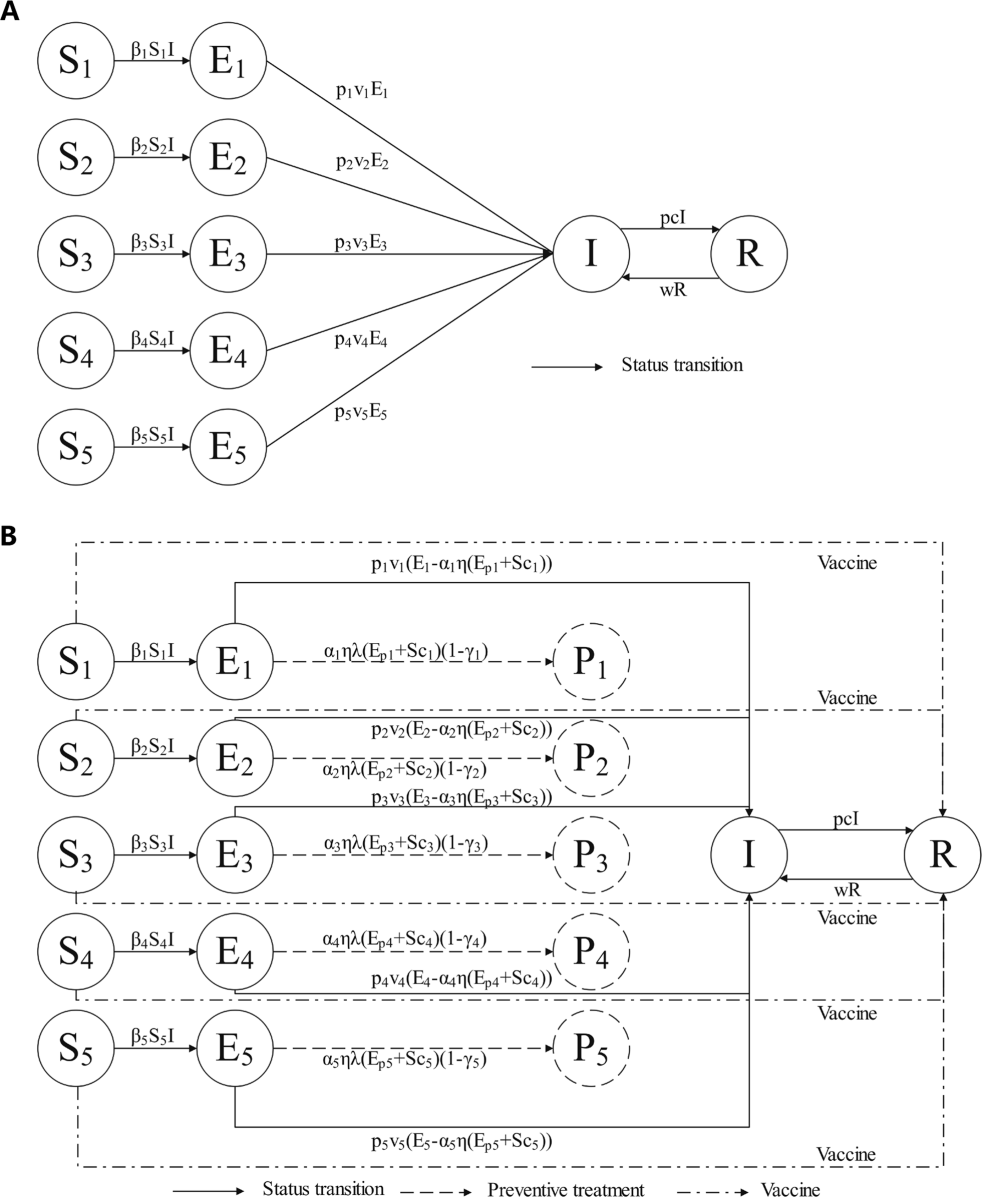
Transmission of TB in five age groups (**A**, Model 1) and with different interventions (**B**, Model 2). β1, β2, β3, β4, β5: TB transmission rate from active TB patients (I) to susceptible people aged 0–4, 5–14, 15–39, 40–64 and 65+ years old; p1, p2, p3, p4, p5: proportion of LTBI patients (**E**) who become active TB patients in each age group; v1, v2, v3, v4, v5: rate at which LTBI patients become active TB patients in each age group; w: relapse rate; p and c: recovery proportion and rate, respectively; Ep1, Ep2, Ep3, Ep4, Ep5: close contacts of different age groups; Sc: LTBIs that are identified by screening (ACF); P1, P2, P3, P4, P5: proportion of people who finished TPT treatment in each age group; η and λ: proportion who completed treatment or a preventive treatment plan, respectively; γ_1_, γ_2_, γ_3_, γ_4_, γ_5_: proportion of failures from a preventive treatment plan in each age group

Two models were developed as extensions of the classical susceptible-exposed-infectious-recovered (SEIR) model to examine the transmission of TB from 2005 to 2035 (Fig. 1). Each model classified susceptible and exposed compartments into five age groups: 0 to 4 years-old (S_1_, E_1_), 5 to 14 years-old (S_2_, E_2_), 15 to 39 years-old (S_3_, E_3_), 40 to 64 years-old (S_4_, E_4_), and 65 years-old and above (S_5_, E_5_).

Model 1 (Fig. 1A) had three major assumptions. First, it assumed the number of people of all ages was evenly distributed for calculation of the number of people who were susceptible and the number who had latent infections of five age groups through the total population and latent infection rate. Second, it assumed to there were no births or deaths during the study period. Third, it assumed that the proportion of recovered patients and the recovery rate remained constant during the study period. Model 1 was initially used to estimate unknown parameters. Then, using these estimated parameters, Model 2 was used to simulate the effects of the preventive treatment and screening for LTBI (Fig. 1B). Table 1, Additional file 1: Tables S1 and S2 define the parameters and provide the initial values.

**Table 1:** Parameters of different preventive treatment regimens

Parameters	Description	1HP	3HP	3HR	4R	6H	9H
η	Completion proportion of TPT	97% [15]	96% [16]	95% [16]	92% [17]	94% [18]	95% [19]
λ	Treatment rate of TPT	1	1/3	1/3	1/4	1/6	1/9
γ_1_	Failure proportion of TPT in LTBIs aged 0–4	1.95% [15]	0.18% [20]	0.25% [21]	0.25% [22]	1% [18]	0.29% [22]
γ_2_	Failure proportion of TPT in LTBIs aged 5–14	1.95% [15]	0.18% [20]	0.25% [21]	0.25% [22]	1% [18]	0.29% [22]
γ_3_	Failure proportion of TPT in LTBIs aged 15–39	1.95% [15]	0.18% [20]	0.25% [21]	0.25% [22]	1% [18]	0.29% [15]
γ_4_	Failure proportion of TPT in LTBIs aged 40–64	1.95% [15]	0.18% [20]	0.25% [21]	0.25% [22]	1% [18]	0.29% [22]
γ_5_	Failure proportion of TPT in LTBIs aged 65+	1.95% [15]	0.18% [20]	0.25% [21]	0.25% [22]	1% [18]	0.29% [22]

### Data analyses

#### Parameters determination

The model was fitted to reported incidence from various sources. The relevant parameters were obtained from basic demographic information, IDRS, TBIMS, and previous publications. A maximum likelihood method was used for parameter estimation. These parameters were used to determine the goodness of fit of the model to the observed data, and a Chi-square test on the number of reported TB cases versus the model fit values was used to examine the goodness of fit. R software version 4.0.5 was used for all statistical analyses, including testing the goodness of fit. Estimation of unknown parameters and simulation of different scenarios were conducted with *deSolve* package [23].

#### Scenarios construction

To construct the scenarios, we used the WHO consolidated guidelines on TB [7] and in the context of China to identify high-risk populations [24] for TPTs. And finally, close contacts of TB patients, HIV-infected patients, juveniles aged 0–14 years, elderly people aged 65 years and above and whole population in areas with incidences greater than 55 per 100,000 were identified as high-risk groups for the implementation of TPTs. In addition to TPTs for high-risk populations, to expand coverage of TPTs and reach the 2035 target, two additional interventions were considered, expansion of close contact tracing capabilities and active cases finding of LTBI. Both were simulated by bringing the population in the E compartments into the P compartments by being tracked by close contacts or by ACF of LTBI. The simulation of the vaccination effect was conducted by moving the vaccinated population from different S compartments to R compartment.

#### Sensitivity analysis

The proportion of patients cured p0, the transmission coefficient β, the proportion of active TB progression p, the relapse rate w, and the patient cure rate c were the five parameters we chose to analyze the sensitivity of Model I’s results. The parameter fluctuation range was set at 0.8 to 1.2 times the original parameter values. To discuss the possible overrepresentation of the preventive treatment completion proportion η, a sensitivity analysis was also performed. The scenario set was the completion of ACF + TPT in elderly population within 4 years. η was set to fluctuate between 0.5 and 1 times its original value.

## Results

### Parameter estimation and model fitting

**Fig. 2:**
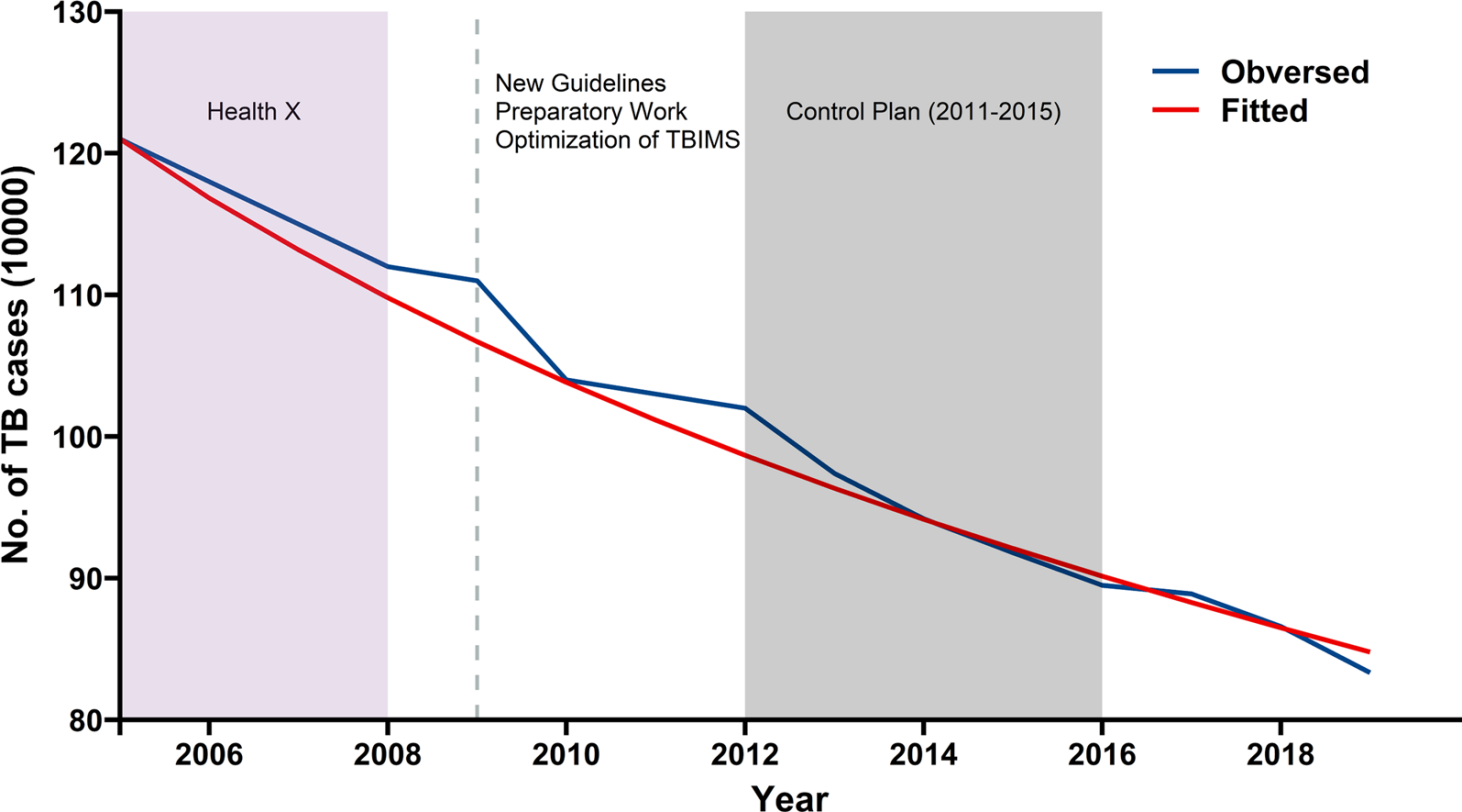
Annual reported TB cases in China from 2005 to 2019 and model results

We initially modeled the incidence of TB in China from 2005 to 2019 with Model 1 using parameter values provided in Additional file 1: Table S1 and initial demographic values provided in Additional file 1: Table S2. Overall, the results indicated the model had a good fit (Fig. 2, χ^2^ = 0.3085, p > 0.999). However, there were some deviations of the model and observed data, especially during 2009 and 2012 when the model provided low estimates. We can suggest several possible reasons for these deviations. First, between 2002 and 2008, China’s National TB Control Program created the “Health X Project”, and this program greatly increased the identification and successful treatment of TB patients [25]. Second, in 2009, the preparatory work of the fifth national TB epidemiological sampling survey was completed, and this helped to improve case detection [26]. Third, in 2009, China completed optimization of the TBIMS and organized related training, which was implemented nation-wide in March; these improved the monitoring and reporting of TB [26]. Fourth, in early 2009, the Ministry of Health issued the “Guidelines for the Implementation of China’s TB Control Plan”, which provided technical support for the standardized implementation of the national TB control program [26]. Fifth, by the end of 2011, the General Office of the State Council issued the “National TB Control Plan (2011–2015)”, whose goal was to identify and treat 4 million patients with pulmonary TB [27].

Through sensitivity analysis of the five parameters selected in Model 1 (Additional file 1: Fig. S1), we found that increasing the proportion of TB patients cured, cure rate of TB patients and decreasing the proportion of active TB progression were able to reduce the incidence of TB more substantially for the same fluctuation range. Reducing the transmission coefficient had a limited effect on the reduction of TB incidence. While the effect of reducing the relapse rate was barely visible due to the already low recurrence rate obtained from the parameter estimates.

### Effect of different TPTs

**Fig. 3:**
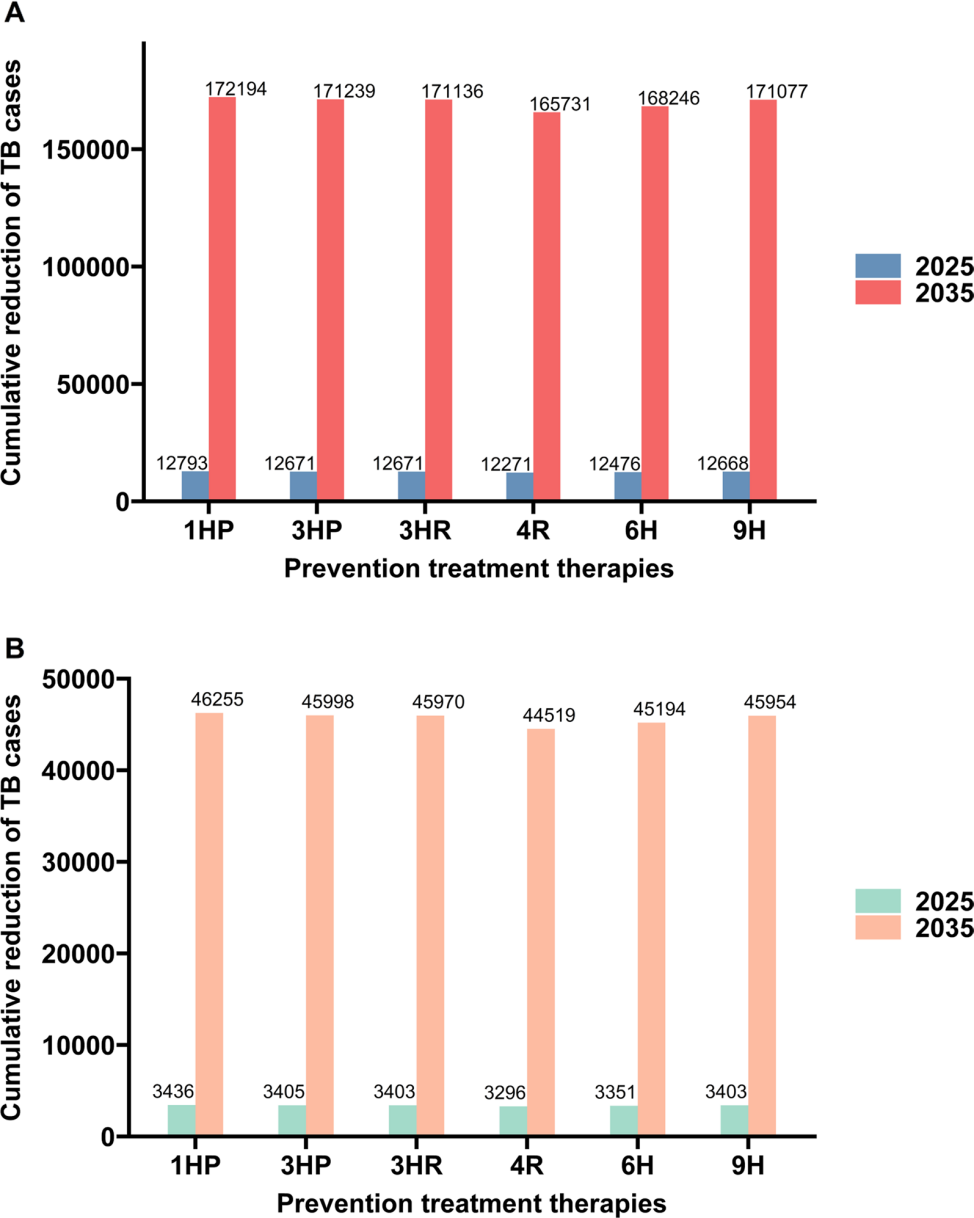
Cumulative reduction in the number of TB cases from 2021 to 2025 and 2035 when all close contacts receive treatment (**A**) and when people with HIV receive treatment (**B**)

We examined the effect of implementing two TPT scenarios beginning in 2021. Scenario 1 assumed that 1,697,248 close contacts (the data were from annual report of Chinese Center for Disease Control and Prevention) were identified each year, and all of them received preventive treatments (Fig. 3A). Scenario 2 assumed that preventive treatments were received by all people infected with Human immunodeficiency virus (HIV) (Fig. 3B).

Compared with no preventive treatment, the 1HP regimen (daily rifapentine [RPT] + isoniazid [INH] for 4 weeks) provided the greatest cumulative decrease in the number of cases by 2035, and this was followed by the 3HP (weekly RPT + INH for 3 months), 3HR (daily rifampin + INH for 3 months), 9H (daily INH for 9 months), 6H (daily INH for 6 months) and 4R (daily RPT for 4 months) regimens. For the first scenario (treatment of all close contacts), the 1HP therapy reduced the total number of TB cases by 12,793 in 2025 and by 172,194 in 2035 (Fig. 3A). For the second scenario (treatment of all people with HIV), 1HP therapy reduced the number of TB cases by 3436 in 2025 and by 46,255 in 2035 (Fig. 3B).

### Effect of ACF for LTBIs and TPT in high-risk populations

According to the effect of different TPT regimens, we only examined scenarios that used the 1HP regimen in subsequent analyses.

**Fig. 4:**
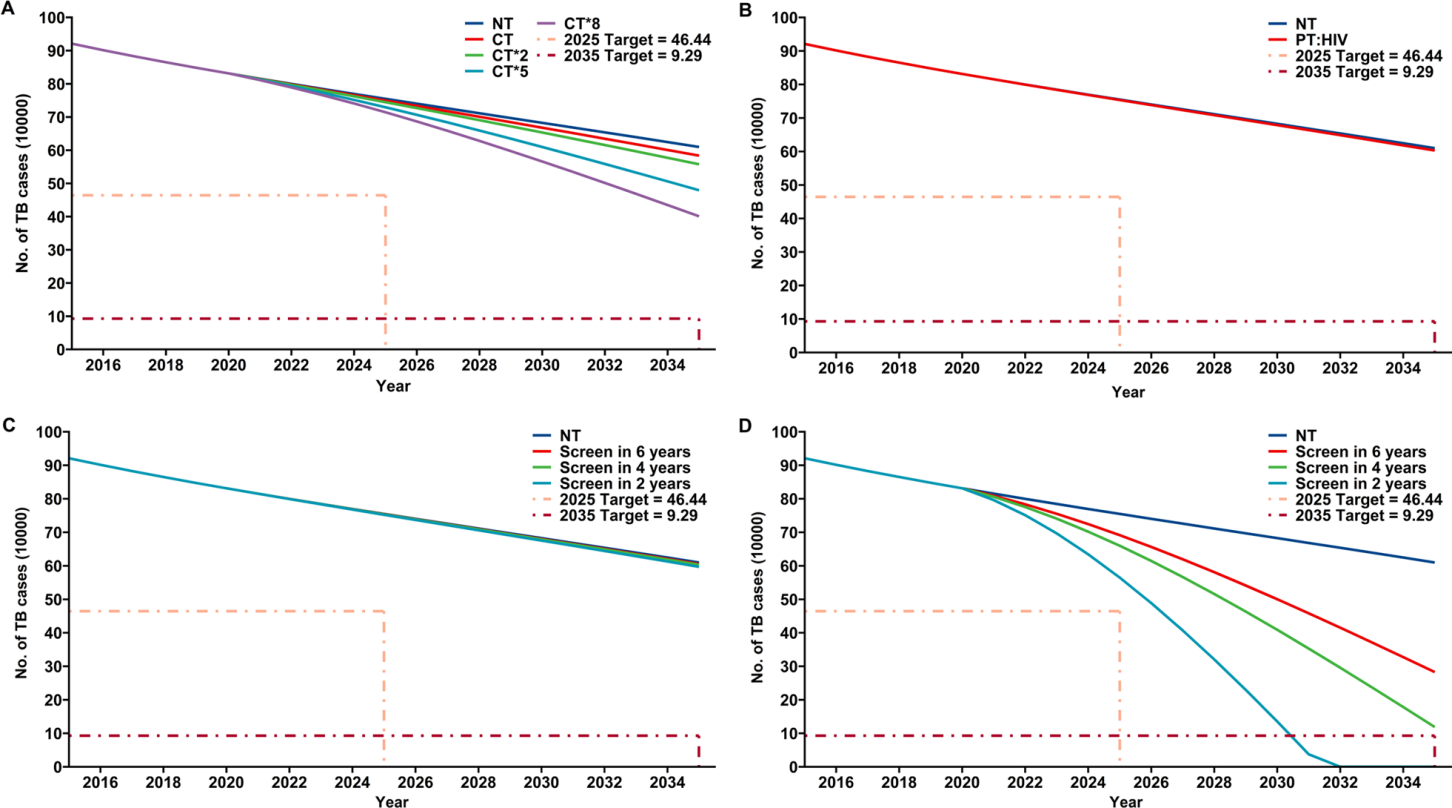
Effect of different ACF strategies for LTBIs on the annual incidence of TB in China from 2021 to 2035. **A** Tracking intensity of close contacts. **B** TPT for HIV infected people. **C** ACF for LTBI in children aged 0 to 14 years. **D** ACF for LTBI in the elderly (> 65 years old). CT: identification of 2 close contacts for each TB patient (baseline); CT*2: twice the baseline (4 contacts), CT*5: five times the baseline (10 contacts); CT*8: eight times the baseline (16 contacts)

Identification of more close contacts: we first compared the number of TB cases for a baseline level of ACF (tracking 2 close contacts per TB patient) with tracking of 4, 10, and 16 close contacts. As expected, an increased intensity of ACF led to fewer TB cases. However, an ACF eight-times the baseline level (16 close contacts per TB patient) did not achieve the 2035 target (Fig. 4A).

Focusing on people infected with HIV: providing all HIV infected people with TPT had a very small effect on the number of TB cases, and the total was well above the 2035 target (Fig. 4B).

Increasing ACF for LTBIs in children: we analyzed the effect of ACF for LTBIs that targeted children and adolescents (age 0–14 years-old), assuming that the screening would be completed within 2, 4, or 6 years (Fig. 4C). Again, these interventions had only a negligible effect on the number of TB cases.

Increasing ACF for LTBIs in the elderly: we analyzed the effect of ACF for LTBIs that targeted the elderly (age of 65 years-old or more), assuming that the screening would be completed within 2, 4, or 6 years. Remarkably, this intervention was highly effective (Fig. 4D). In particular, completion of this screening within 2 years achieved the 2035 target ahead of schedule (2031), although the 2025 target was not achieved (with an 11.3% excess of cases).

### Effectiveness of a new vaccine against TB

**Fig. 5:**
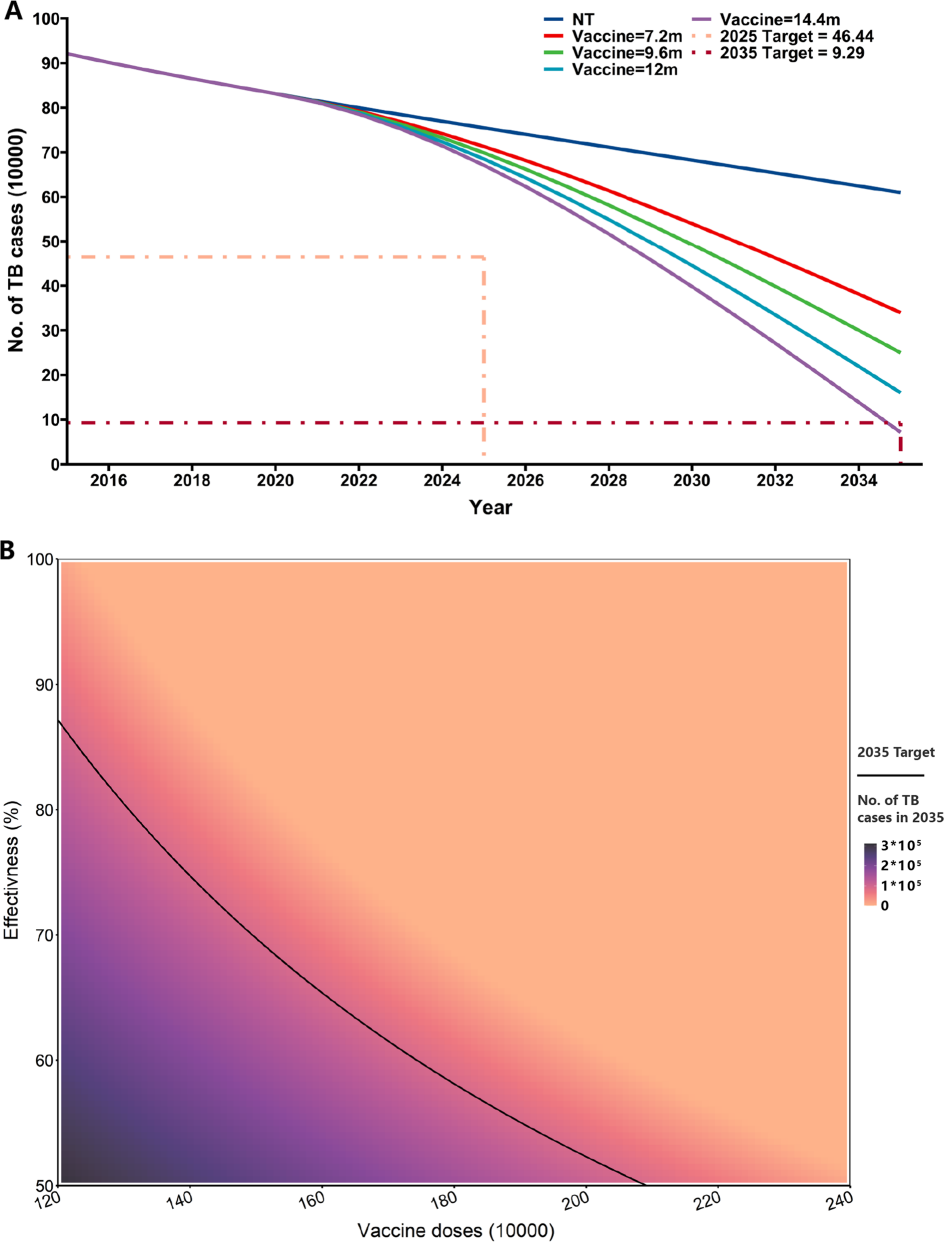
Effect of a new ANTI-TB vaccine on the incidence of tuberculosis from 2021 to 2035. **A** Changes in the number of TB cases after vaccination, assuming 100% vaccine effectiveness. **B** Number of TB cases in 2035 for lower levels of vaccine effectiveness and vaccination doses, the black line denotes the target for 2035

We examined the impact of a new anti-TB vaccine that was introduced in 2021 and initially assumed it was 100% effective. The current Bacillus Calmette–Guérin (BCG) vaccine already provides protection to children aged 0 to 14 years. And there has been a growing tendency to focus on adolescents and adults [10]. Thus, we examined the effect of 7.2 million, 9.6 million, 12 million, and 14.4 million annual doses of vaccines for the other three age groups. This intervention led to large decreases in the number of active TB cases (Fig. 5A). When there were 14.4 million annual vaccine doses, the 2035 target of a 90% reduction in the incidence of TB was achieved.

Since a vaccine with 100% effectiveness is not achievable and the more effective vaccines currently available have an efficacy rate of 49.7% [28]. We also examined the effects of using a vaccine that had lower effectiveness and the effects of different rates of vaccine coverage (Fig. 5B). These results indicated that the 2035 target was reached when the product of vaccine effectiveness and the number of annual inoculations in each age group was 10.5 million.

### Effect of ACF for LTBIs and TPT in areas with different TB burdens

**Fig. 6:**
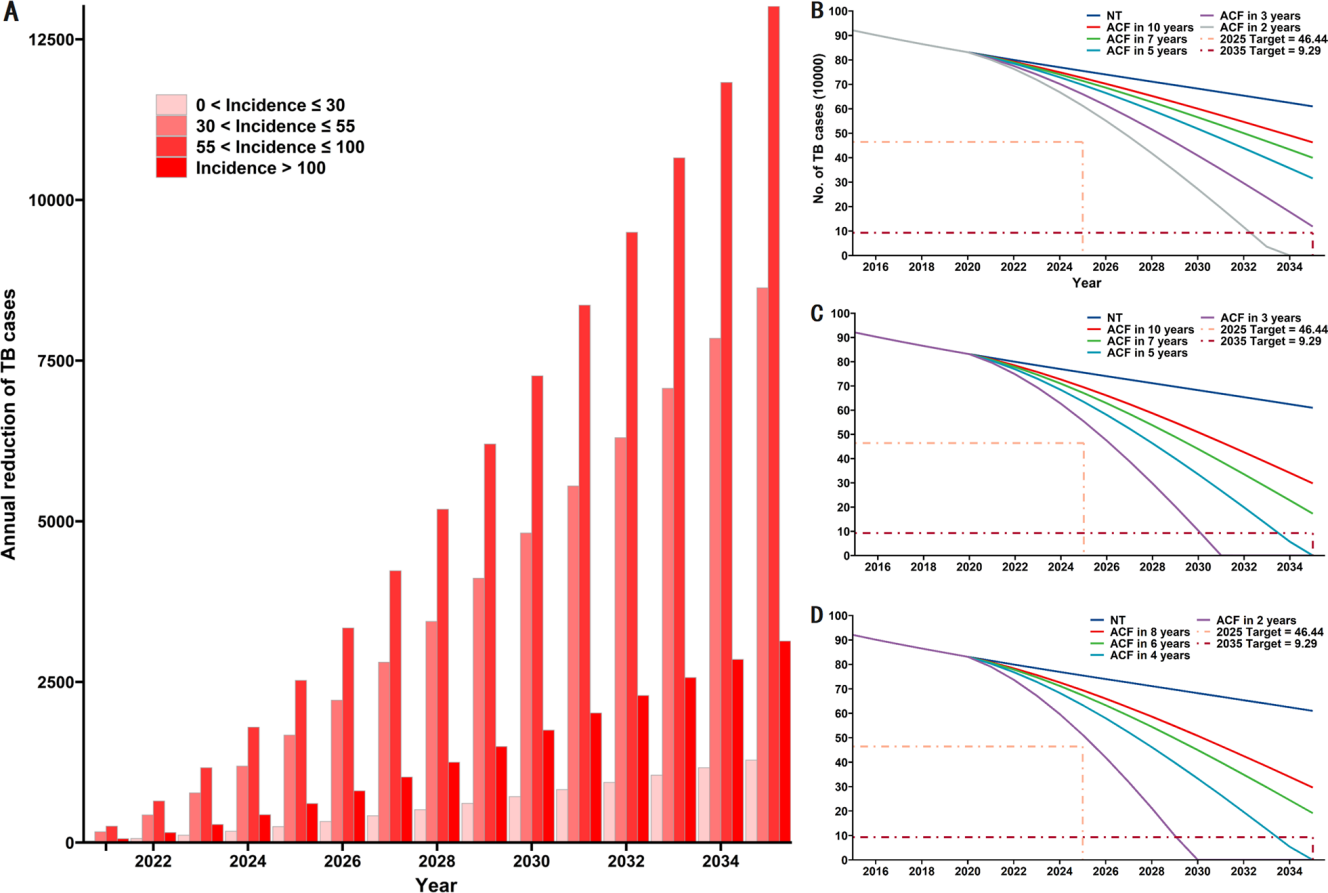
Impact of different ACF and TPT strategies for LTBIs on the number of TB cases from 2021 to 2035. **A** Preventive treatment for people in close contact with TB in regions with different incidences (lower than 30 per 100,000 to above 100 per 100,000). **B** ACF for LTBIs and TPT in areas with incidences greater than 55 per 100,000. **C** ACF for LTBIs and TPT at a national scale. **D** ACF for LTBIs and TPT in elderly and areas with incidences greater than 55 per 100,000

We then examined the effects of preventive treatments in areas with different incidences of TB. The cumulative reduction in the number of TB cases was greatest when areas with incidences between 55 to 100 per 100,000 were targeted (Fig. 6A). We therefore examined the effect of ACF for LTBIs and TPT in the areas with incidences greater than 55 per 100,000. These results indicated that completion of ACF for LTBIs in areas with high TB incidence within 2 years achieved the 2035 target ahead of schedule (2033), although this intervention did not achieve the 2025 target (Fig. 6B). When the ACF for LTBIs is introduced at a nation-wide level and all patients with LTBIs received TPT, the annual incidence of TB decreased at an even faster rate (Fig. 6C). In particular, when this nation-wide ACF for LTBIs was completed in 3 years, the 2035 target was achieved in 2030; if the nation-wide ACF for LTBIs was completed in 5 years, this target was achieved in 2034. However, completion of a nation-wide ACF for LTBIs within 3 years did not achieve the 2025 target.

To study the effect of the interventions on multiple populations, we selected the elderly population and the whole population in areas with high TB burden, the two most effective high-risk populations, for simulation. The results are shown in Fig. 6D, when ACF for LTBIs was completed in 4 years, the 2035 target was achieved before 2034. It’s worth noting that when ACF for LTBIs was completed in 2 years, the incidence of TB would decline rapidly and was even on track to reach the 2025 target of “End TB Strategy” by 2026.

## Discussion

We used a SEIR model to simulate the effect of different strategies on the prevention and control of TB in China. Among the six preventive treatments, 1HP was the most effective, in that it led to the smallest number of TB cases by 2035. It is likely that this treatment had the best performance because it has the shortest duration, provides the fastest recovery, and has high patient compliance [15].

Without vaccination, use of a nation-wide ACF and TPT for LTBIs, focusing on high incidence areas, and focusing on the elderly were the most effective strategies. The 2035 target of the “End TB Strategy” was achieved when nationwide ACF and TPT are completed within 5 years, when ACF and TPT were completed in high incidence areas within 2 years, when TPT in the elderly was completed within 2 years or when ACF and TPT were completed in both of the above populations within 4 years. A more rapid completion of the ACF and TPT for LTBIs further reduced the cumulative number of TB cases. Since the source of the parameters we used for TPT simulations was studies in Africa or on HIV patients. And the completion proportion of TPT may be higher than if it was conducted on a large scale in China. We performed a sensitivity analysis on the proportion of completion (Additional file 1: Fig. S2). The results suggested that a reduction in the proportion of TPT completion can affect the effect of this intervention substantially. In the scenario of completing ACF + TPT for the elderly population within 4 years, the number of reported TB cases would be 2.07 times higher when the proportion of completion is reduced to 50% of the original. It can be seen that in addition to the high-intensity LTBI screening and TPT intervention for high-risk groups, maintaining the completion proportion of TPT is another key to ensure the effectiveness of the intervention.

Due to the protective effect of the BCG vaccine in children and adolescents, TB has a relatively low incidence in Chinese children. Thus, the ACF and TPT for LTBIs only provide a small benefit for this age group. Previous study has also suggested that the introduction of tuberculosis vaccine targeting adolescents has little impact on the epidemic of tuberculosis in China. On the contrary, delivering new post-infection vaccines to older adults can be crucial to provide an important contribution to attaining WHO goals [10]. However, children have many contacts at school, in the family, and in the community, and can therefore have significant roles in the transmission of TB [12]. Therefore, TB surveillance in children and adolescents is essential for prevention and control of TB, especially in areas with low incidence. Individuals infected with HIV have a higher risk of TB [13], and TB is the leading cause of death in people with HIV [14]. Although the effects of TPT in HIV patients seem to be limited, this intervention remains essential for reducing overall mortality from TB in people with HIV [29, 30].

We also evaluated the effect of a hypothetical anti-TB vaccine for people who were at least 14 years-old. Based on previous assessments of vaccine efficiency [28], we found that the 2035 target can be achieved when the product of annual vaccination doses and vaccine efficiency reached 10.5 million in each age group. Therefore, it is necessary to attain a high rate of coverage when a new and effective vaccine becomes available. Although there has been progress in the development of new anti-TB vaccines [31], the effectiveness of existing vaccines are insufficient for controlling the TB epidemic in China.

There are a huge number of people with LTBIs in China [9]. Thus, even the identification of 16 close contacts per patient intense ACF was insufficient to achieve the 2035 target. In other words, simply relying on the expansion of ACF will not achieve the 2035 target.

The COVID-19 pandemic has also resulted in a halt in TB control in China. In each given month in 2020, the number of TB reports is lower than the average for 2019 [1]. The availability of TB detection and treatment services has drastically declined, as has the capacity to identify new TB patients. For all of these reasons, achieving the target of WHO’s “End TB Strategy” is becoming increasingly challenging. China can only prepare for the elimination of TB by resuming the original control measures as soon as feasible and expanding the deployment of novel intervention.

Considering the different scenarios involved in the models, we compared the predictive efficiency until 2019 of other model studies of tuberculosis conducted in a Chinese context. We found that our fit to the available incidence data seemed to have better efficiency compared to the study by Wang et al. [32] But the estimated incidence was slightly higher than the dynamic model constructed by Liu et al. [33] Based on the results of the combined analysis and calibration of 11 mathematical models by Houben et al. [34] we had very similar comparison of the trends in incidence rates from 2005 to 2010. Their study also found that their included model studies only used ACF for LTBIs and TPTs as a general description and did not examine the probably effect of these interventions through the model. And comparing the Markov model constructed by Li et al. [35], we have similar results for the estimated change in overall incidence of TB in China after 2019.

In this study, we developed a SEIR model that considered preventive treatment and innovative vaccines based on the age composition of China, and we discovered that ACF for LTBIs and TPT for TB control in China’s elderly population and high disease burden areas generated excellent effect. We think that the model is ideally adapted to analyzing the impacts of a novel treatment or vaccination that is widely used in China (the same applies to interventions with different effects in different age groups). For countries with a high TB burden, our study also provides inspiration for the conduct of TPT. First, even though various TPT regimens have a good protective effect. The key to achieving a significant reduction in TB incidence is to improve the coverage of TPT through ACF for LTBI. Second, ACF + TPT has good efficiency in elderly population and areas with high TB burden, which should be considered as priority populations for intervention. Finally, interventions for close contacts and HIV-infected populations may not necessarily lead to a significant reduction in TB incidence, but given their great risk, interventions for such populations should be maintained. However, it is important to note that the findings of the model may not be suitable for directly generalizing in other high TB burden countries. Because differences in the age structure of the population and regional differences in incidence may affect the results of the model.

In addition, there are certain limitations. First, the TB data obtained in this study was insufficient. The current model is for a national scale study in China. But it was impossible to discern between different age groups or different types of TB cases based on the IDRS incidence data, which resulted in a decline in model fitting efficiency. Also, possible underreporting and misreporting in IDRS and TBIMS may lead to overestimation or underestimation of the effectiveness of existing measures to control TB in China in the model. Second, due to the aforementioned issues, the current data does not enable for the separation of different age groups creating discrete I compartment, leading to lower accuracy of predicting the cure rate for various age groups of the incidence population. Third, the study may have exaggerated the completion proportion of TPT and could not quantify the impact of drug-resistant TB on disease transmission and cure rate due to the difficulty to correctly identify between patients with drug-resistant TB and drug-sensitive TB in the data obtained. Finally, phase III clinical trials of the efficacy and safety of immunotherapy for TPT have also been mentioned in recent years in the WHO’s TB reports [36, 37]. However, only WHO-recommended regimens were evaluated in this study because we lacked the relevant complete parameters of immunotherapy.

## Conclusions

The overall incidence of TB in China decreased by 9.2% from 2015 to 2019. Despite this progress, interventions that go beyond the present preventive interventions and strategies are needed to reach the 2025 and 2035 targets of the “End TB Strategy” [34, 38]. Introduction of a new tuberculosis vaccine, implementing ACF and TPT for LTBIs at a nation-wide scale, in areas with high TB incidence, and in the elderly could allow China to attain the 2035 target.

## Supplementary Information

**Additional file 1: Figure S1.** Sensitivity analysis of Model I. The proportion of patients cured p0 (A), the transmission coefficient β (B), the proportion of active TB progression p (C), the relapse rate w (D), and the patient cure rate c (E). **Figure S2.** Sensitivity analysis of preventive treatment completion rate η. **Table S1.** Model parameters values used to fit and simulate. **Table S2.** Initial demographic values used to fit and simulate.

## Data Availability

All aggregated parameters and demographic data analyzed in this study are included in the article and its Additional file 1: Tables S1 and S2. Annual incidence data on active TB cases from 2005 to 2019 are available on GitHub at https://github.com/wwbgroup/TB_Incidence-data. All data supporting the findings of this study are available from the authors upon request.

## Abbreviations

TB: Tuberculosis
Mtb: *Mycobacterium tuberculosis*
LTBI: Latent tuberculosis infection
WHO: World Health Organization
IDRS: Infectious diseases report system
TBIMS: Tuberculosis information management system
ACF: Active case-finding
HIV: Human immunodeficiency virus
1HP: A daily dose of rifapentine plus isoniazid for 1 month
3HP: A weekly dose of rifapentine and isoniazid for 3 months
3HR: A daily dose of rifampicin plus isoniazid for 3 months
4R: A daily dose of rifampicin for 4 months
6H: A daily dose of isoniazid for 6 months
9H: A daily dose of isoniazid for 9 months
