# Supplementary material for: Effect of different interventions for latent tuberculosis infections in China: a model-based study
Source: BMC Infect Dis. 2022 May 23;22:488. doi: 10.1186/s12879-022-07465-5 (PMC9125978; doi:10.1186/s12879-022-07465-5)
Supplement: Supplementary file 1 — Additional file 1: Figure S1. Sensitivity analysis of Model I. The proportion of patients cured p0 (A), the transmission coefficient β (B), the proportion of active TB progression p (C), the relapse rate w (D), and the patient cure rate c (E). Figure S2. Sensitivity analysis of preventive treatment completion rate η. Table S1. Model parameters values used to fit and simulate. Table S2. Initial demographic values used to fit and simulate. [file 12879_2022_7465_MOESM1_ESM.docx]

**Additional file for**

**Title:** **E****ffect of different interventions for latent tuberculosis infections in China: a model-based study**

Zexuan Wen^1†^, Tao Li^3†^, Wenlong Zhu^1,4†^, Wei Chen^3^, Hui Zhang^3*^, Weibing Wang^1,2,4*^

1 Department of Epidemiology, School of Public Health, Fudan University, Shanghai 200032, China

2 Key Laboratory of Public Health Safety of Ministry of Education, Fudan University, Shanghai 200032, China

3 National Center for Tuberculosis Control and Prevention, Chinese Center for Disease Control and Prevention, Beijing 100050, China

4 Shanghai Institute of Infectious Disease and Biosecurity, Fudan University, Shanghai 200032, China

^†^ZW, TL and WZ contributed equally to this work.

^*^ Corresponding author:

Dr. Weibing Wang

Affiliation: School of Public Health, Fudan University

Email: wwb@fudan.edu.cn

Dr. Hui Zhang

Affiliation: National Center for Tuberculosis Control and Prevention, Chinese Center for Disease Control and Prevention

Email: [zhanghui@chinacdc.cn](mailto:zhanghui@chinacdc.cn)
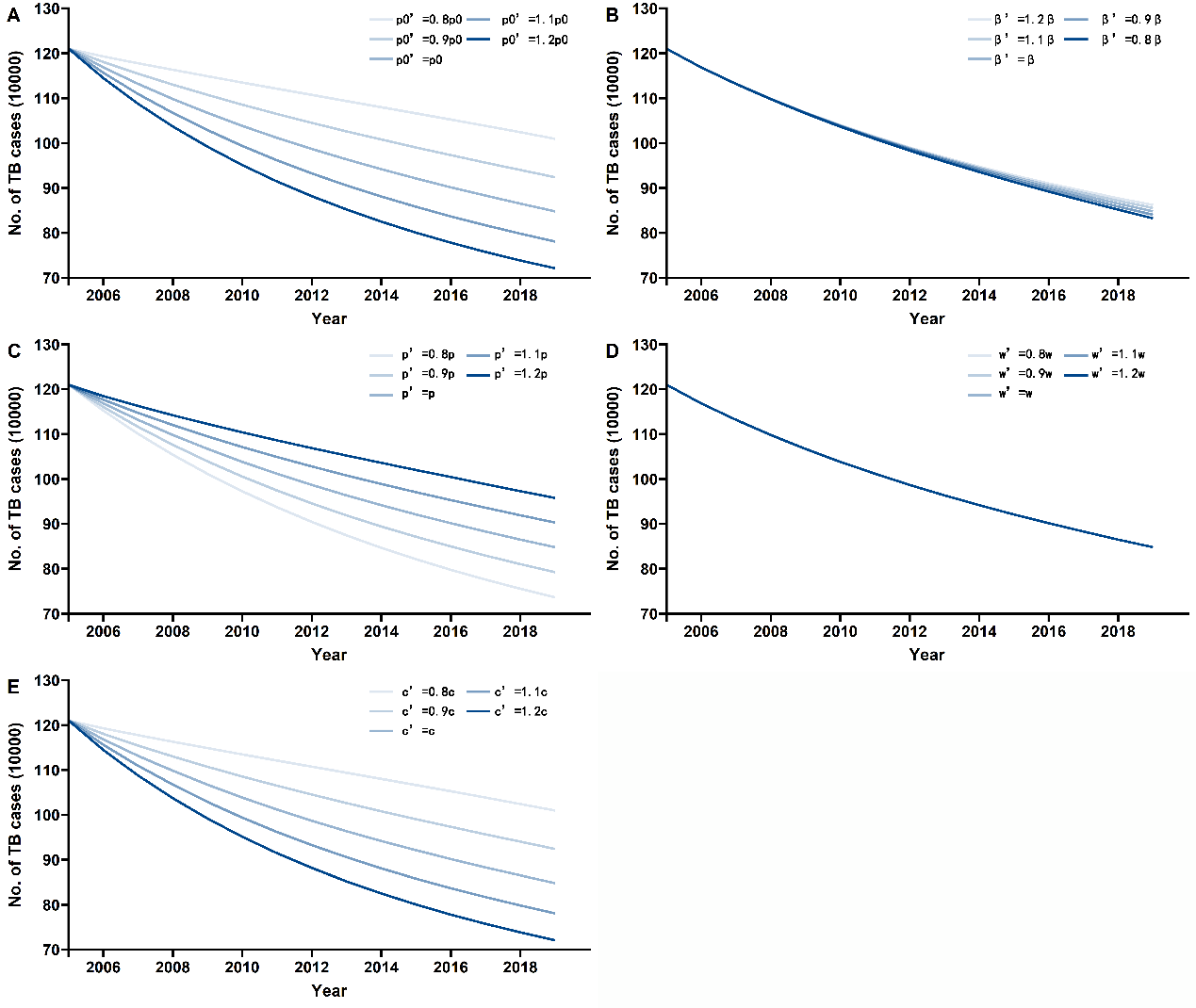


**Figure S1.Sensitivity analysis of Model Ⅰ.** **The proportion of patients cured p0** **(A), the transmission coefficient β (B), the proportion of active TB progression p (C), the relapse rate w (D), and the patient cure rate c (E)**


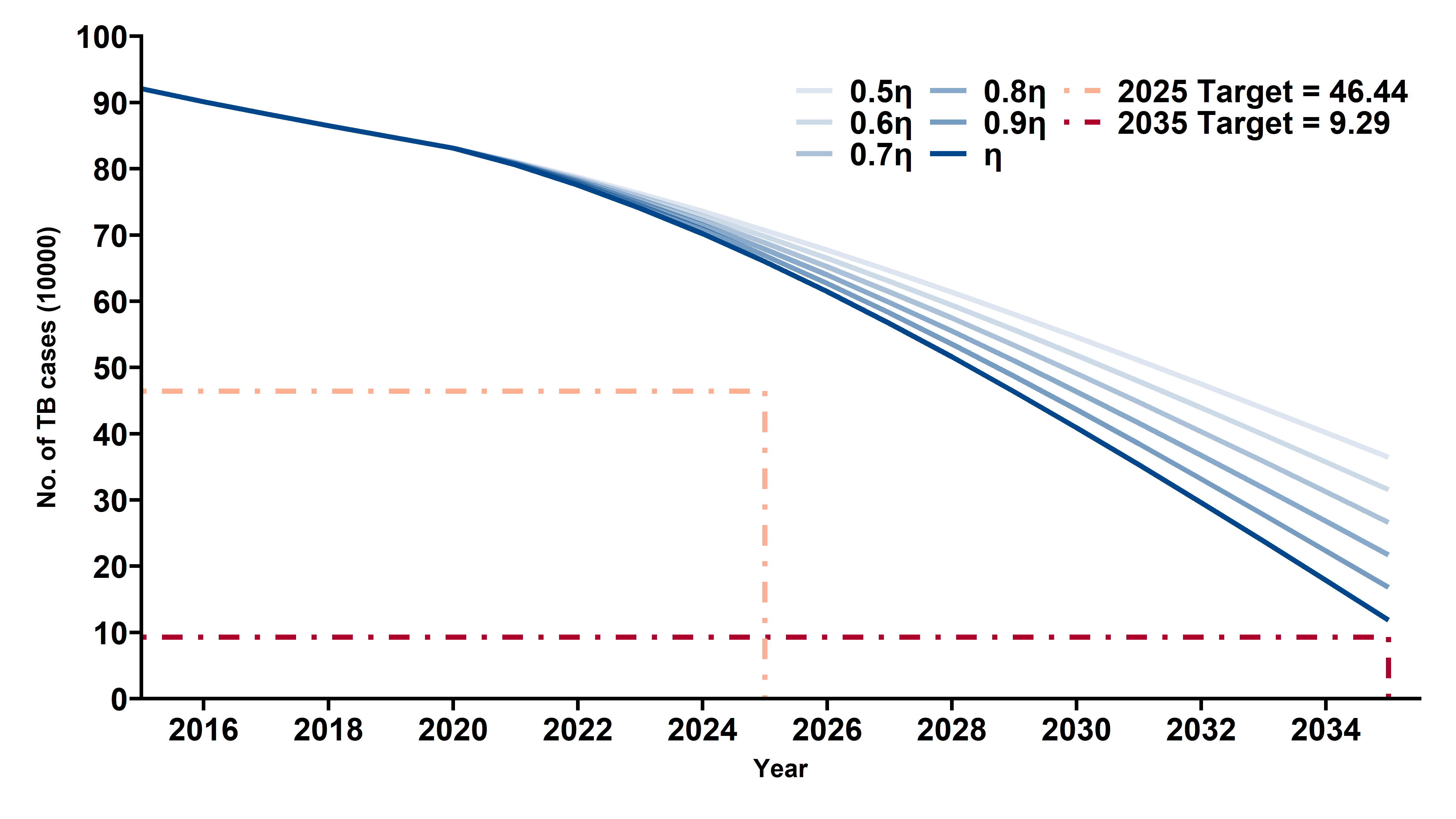


**Figure S2 Sensitivity analysis of** **preventive treatment completion rate η**

**Table S1. Model parameters values used to fit and simulate.**

| Parameters | Description | Parameter value | Source |
| --- | --- | --- | --- |
| β1 | Transmission rate from active TB patients to susceptible people aged 0-4 | 1.2498*10^-5^ | Estimated/Maximum likelihood |
| β2 | Transmission rate from active TB patients to susceptible people aged 5-14 | 9.1022*10^-8^ | Estimated/Maximum likelihood |
| β3 | Transmission rate from active TB patients to susceptible people aged 15-39 | 5.7129*10^-8^ | Estimated/Maximum likelihood |
| β4 | Transmission rate from active TB patients to susceptible people aged 40-64 | 3.2908*10^-5^ | Estimated/Maximum likelihood |
| β5 | Transmission rate from active TB patients to susceptible people aged 65+ | 1.9949*10^-5^ | Estimated/Maximum likelihood |
| p1 | Proportion of LTBI patients aged 0-4 become active TB patients in | 0.022 | Ref 1–4 |
| p2 | Proportion of LTBI patients aged 5-14 become active TB patients in | 0.022 | Ref 1–4 |
| p3 | Proportion of LTBI patients aged 15-39 become active TB patients in | 0.17 | Ref 1–4 |
| p4 | Proportion of LTBI patients aged 41-64 become active TB patients in | 0.17 | Ref 1–4 |
| p5 | Proportion of LTBI patients aged 65+ become active TB patients in | 0.28 | Ref 1–4 |
| v1 | Rate of LTBI patients aged 0-4 become active TB patients | 1.2500*10^-5^ | Ref 1,5 |
| v2 | Rate of LTBI patients aged 5-14 become active TB patients | 1.2500*10^-5^ | Ref 1,5 |
| v3 | Rate of LTBI patients aged 15-39 become active TB patients | 1.9167*10^-5^ | Ref 1,5 |
| v4 | Rate of LTBI patients aged 40-64 become active TB patients | 1.9167*10^-5^ | Ref 1,5 |
| v5 | Rate of LTBI patients aged 65+ become active TB patients | 1.6000*10^-4^ | Ref 1,5 |
| w | Relapse rate | 1.8690*10^-6^ | Estimated/Maximum likelihood |
| p | Proportion of active TB recover | 0.85 | Ref 6 |
| c | Recovery rate | 0.126 | Computing according to treatment period of DS-TB and DR-TB |

**Table S2. Initial demographic values used to fit and simulate**

| Parameters | Description | Parameter value | Source |
| --- | --- | --- | --- |
| S1(0) | Initial number of susceptible people aged 0-4 | 37,992,992 | Ref ^7^, computing |
| S2(0) | Initial number of susceptible people aged 5-14 | 101,101,202 | Ref ^7^, computing |
| S3(0) | Initial number of susceptible people aged 15-39 | 280,109,383 | Ref ^7^, computing |
| S4(0) | Initial number of susceptible people aged 40-64 | 227,673,362 | Ref ^7^, computing |
| S5(0) | Initial number of susceptible people aged 65+ | 64,388,873 | Ref ^7^, computing |
| E1(0) | Initial number of LTBIs aged 0-4 | 30,462,850 | Ref ^7,8^, computing |
| E2(0) | Initial number of LTBIs aged 5-14 | 81,063,126 | Ref ^7,8^, computing |
| E3(0) | Initial number of LTBIs aged 15-39 | 224,592,208 | Ref ^7,8^, computing |
| E4(0) | Initial number of LTBIs aged 40-64 | 182,548,912 | Ref ^7,8^, computing |
| E5(0) | Initial number of LTBIs aged 65+ | 51,627,114 | Ref ^7,8^, computing |
| I(0) | Initial number of active tuberculosis patients | 108,333 | Statutory infectious disease report |
| R(0) | Initial number of recovery people | 0 | Assumption |

**REFERENCES**

1. Harris RC, Sumner T, Knight GM, Evans T, Cardenas V, Chen C, et al. Age-targeted tuberculosis vaccination in China and implications for vaccine development: a modelling study. Lancet Glob Health. 02 2019;7(2):e209–18.

2. Knight GM, Griffiths UK, Sumner T, Laurence YV, Gheorghe A, Vassall A, et al. Impact and cost-effectiveness of new tuberculosis vaccines in low- and middle-income countries. Proc Natl Acad Sci U S A. 2014 Oct 28;111(43):15520–5.

3. Abu-Raddad LJ, Sabatelli L, Achterberg JT, Sugimoto JD, Longini IM, Dye C, et al. Epidemiological benefits of more-effective tuberculosis vaccines, drugs, and diagnostics. Proc Natl Acad Sci U S A. 2009 Aug 18;106(33):13980–5.

4. Dye C, Watt CJ, Bleed DM, Williams BG. What is the limit to case detection under the DOTS strategy for tuberculosis control? Tuberculosis (Edinb). 2003;83(1–3):35–43.

5. Ozcaglar C, Shabbeer A, Vandenberg SL, Yener B, Bennett KP. Epidemiological models of Mycobacterium tuberculosis complex infections. Math Biosci. 2012 Apr;236(2):77–96.

6. WHO. Global tuberculosis report 2020. Geneva. 2020.

7. National Bureau of Statistics of China. China Statistical Yearbook 2020. Beijing: China Statistics Press; 2020.

8. The Fifth National Tuberculosis Epidemiological Sampling Survey Technical Guidance Group, The Fifth National Tuberculosis Epidemiological Sampling Survey Office. Report on the Fifth National Tuberculosis Epidemiological Survey in 2010. Chinese Journal of Antituberculosis 2012; 34: 485–508.
